# Supplementary material for: Influence of RNA circularity on Target RNA-Directed MicroRNA Degradation
Source: Nucleic Acids Res. 2024 Feb 21;52(6):3358–74. doi: 10.1093/nar/gkae094 (PMC11014252; doi:10.1093/nar/gkae094)
Supplement: gkae094_supplemental_files [file gkae094_supplemental_files.zip › NAR-02331-Q-2023.R1_Supplementary Figures.pdf]

# Supplementary Figure 1

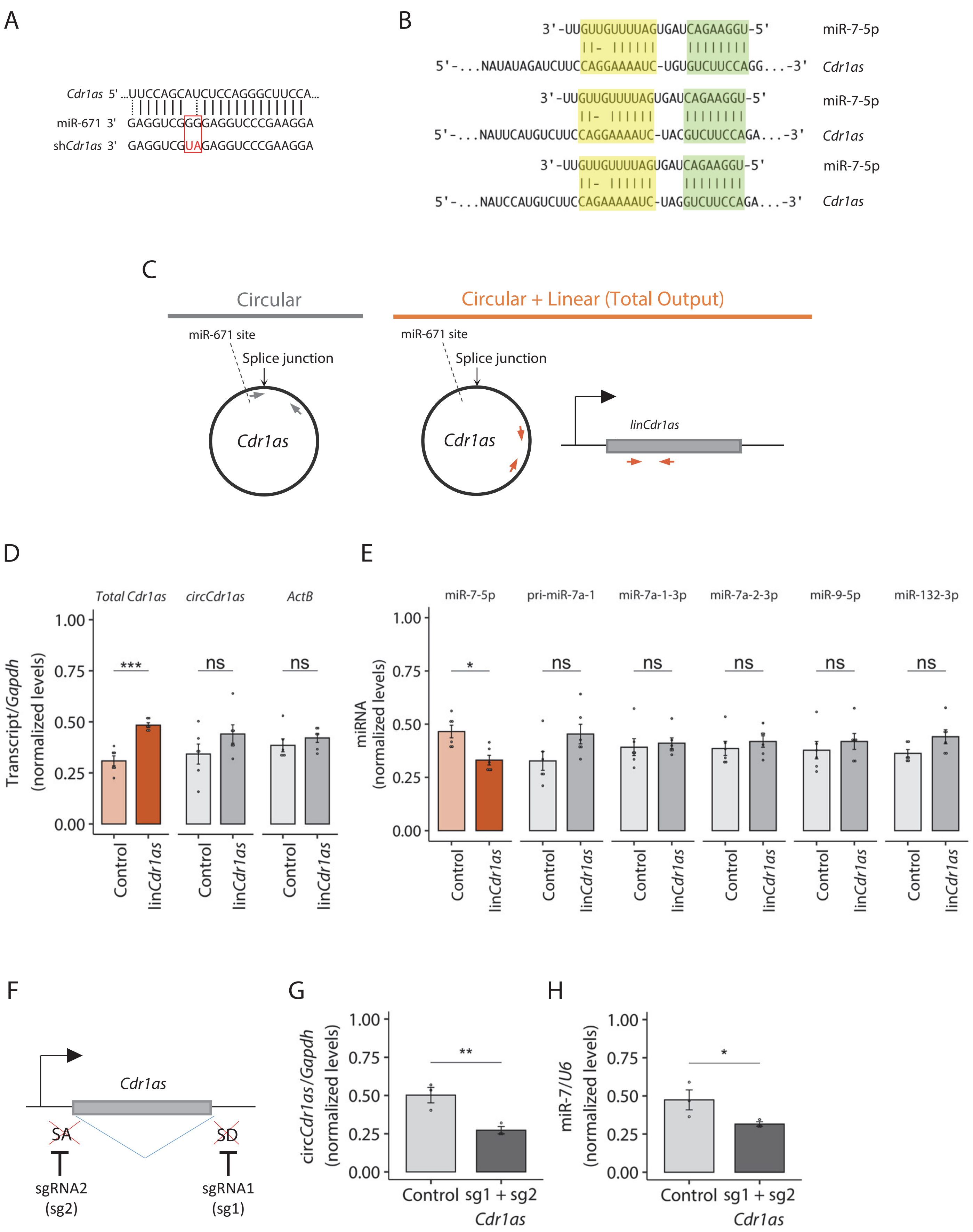

**Supplementary Figure 1.**

**MicroRNA site illustrations, quality controls and primer design for *Cdr1as*.**

A Scheme depicting the sequences of the miR-671 binding site within *Cdr1as*, miR-671 and the engineered sh*Cdr1as*. The latter is based on miR-671 with two nucleotide changes that make it fully complementary to the circRNA to maximize its slicing capacity.

B Three examples of potential TDMD-competent binding sites for miR-7 present in *Cdr1as*, aligned and illustrated using scanMiR.

C Illustration of the primer designs for measuring the different *Cdr1as* isoforms (detailed in Materials and Methods).

D Expression levels of *Cdr1as* total output, circular *Cdr1as* and unrelated gene (*Actb*) upon over-expression of linear *Cdr1as* (lin*Cdr1as*). Levels were normalized to *Gapdh*. n = 6 culture wells (from 2 independent primary cultures) for each condition.

E Expression levels of miR-7 guide strand (miR-7-5p), passenger strands (miR-7a-1-3p and miR-7a-2-3p), primary RNA (pri-miR-7a-1) and two unrelated miRNAs (miR-9-5p and miR-132-3p). All miRNAs levels were normalized to *U6*, while the pri-miR level was normalized to *Gapdh*. n = 6 culture wells (from 2 independent primary cultures) for each condition.

F Illustration of the strategy designed to mutate *Cdr1as* splicing sites by CRISPR/Cas9.

G *Cdr1as* total output levels upon CRISPR/Cas9 editing of the *Cdr1as* splicing sites, measured by RT-qPCR in primary hippocampal neurons. Control corresponds to a transduced linear transcript (GFP-expressing). n = 3 culture wells (from 1 independent primary culture) for each condition.

H MiR-7 abundance measured by Taqman RT-qPCR in the same samples as in F. n = 3 culture wells (from 1 independent primary culture) for each condition.

Data are presented as mean ± SEM. Statistical significance was determined by unpaired Student's *t tests* (ns: p > 0.05, *: p <= 0.05, **: p <= 0.01, ***: p <= 0.001, ****: p <= 0.0001). In panels C and D, Bonferroni's correction for multiple testing was applied. For the F and G panels, equal variance was assumed.

# Supplementary Figure 2

A

| Construct purpose | miR-132 sites: Type | miR-132 sites: Architecture | miR-124 sites: Type | miR-124 sites: Architecture | Expected RNA topology |
|---|---|---|---|---|---|
| circRNA expression (linear RNA-downregulated) | Bulged (WT) | GCUGGUACCG ACAT CUGACAAU-5' miR-132 / CGACCAUGGC NNN GACUGUUA target | Perfect match | CCGUAAGUGGCGCACGGAAU-5' miR-124 / GGCAUUCACCGCGUGCCUUA target | Circular + low linear leak |
| | Seed-mut | GCUGGUACCG ACAT CUG GACA U-5' miR-132 / CGACCAUGGC NNN GAC GAAC A target | Perfect match | CCGUAAGUGGCGCACGGAAU-5' miR-124 / GGCAUUCACCGCGUGCCUUA target | Circular + low linear leak |
| circRNA expression (control) | Bulged (WT) | GCUGGUACCG ACAT CUGACAAU-5' miR-132 / CGACCAUGGC NNN GACUGUUA target | Seed-mut | CCGUAAGU GGCG CA CGGA AU-5' miR-124 / GGCAUUCA NNN GU CGGA UA target | Circular + high linear leak |
| | Seed-mut | GCUGGUACCG ACAT CUG GACA U-5' miR-132 / CGACCAUGGC NNN GAC GAAC A target | Seed-mut | CCGUAAGU GGCG CA CGGA AU-5' miR-124 / GGCAUUCA NNN GU CGGA UA target | Circular + high linear leak |
| linear RNA expression (TDMD inducer) | Bulged (WT) | GCUGGUACCG ACAT CUGACAAU-5' miR-132 / CGACCAUGGC NNN GACUGUUA target | NA | NA | Linear |
| | Seed-mut | GCUGGUACCG ACAT CUG GACA U-5' miR-132 / CGACCAUGGC NNN GAC GAAC A target | NA | NA | Linear |

B

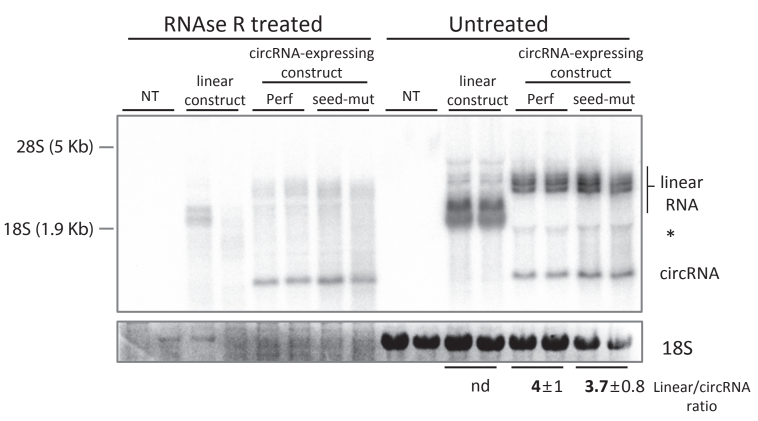


C

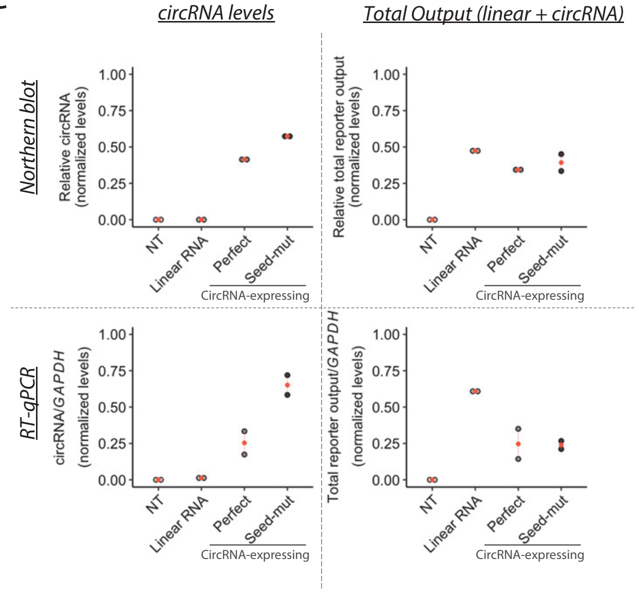


D

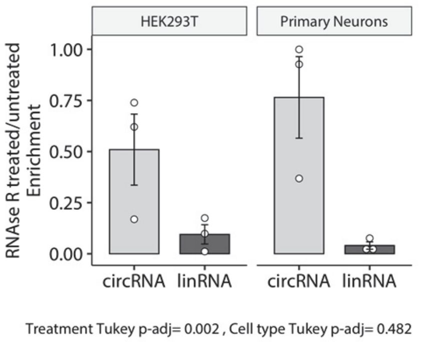


E

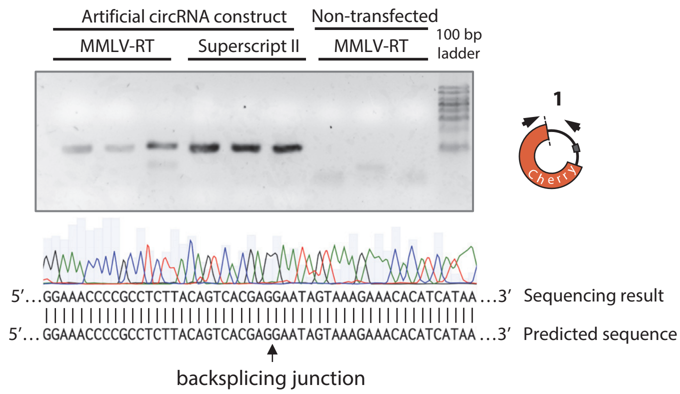


F

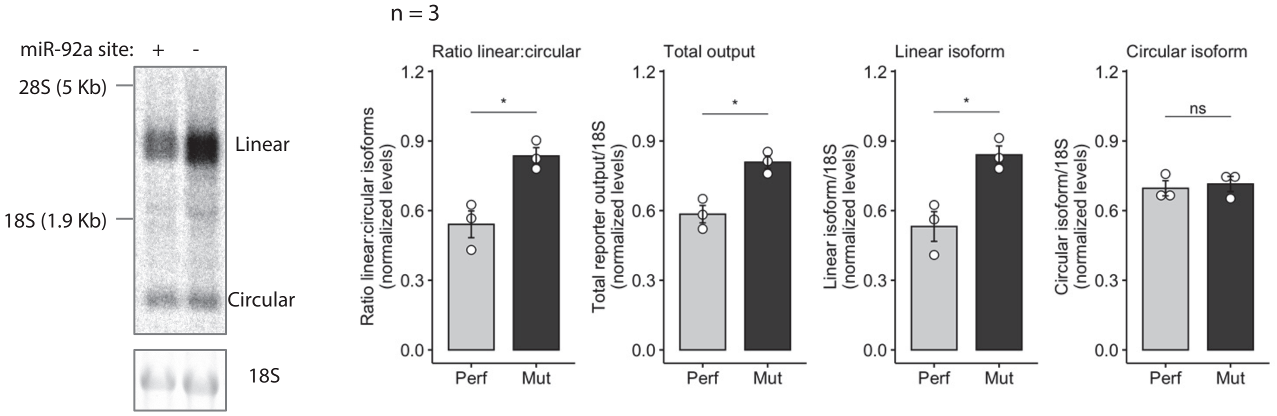

**Supplementary Figure 2.**

**CircRNA-expressing constructs and quality controls.**

A Table summarizing the generated constructs.

B Northern blot analysis of RNase R treated or untreated samples from HEK293T cells expressing the indicated constructs with either perfect or seed-mutant miR-124 binding sites. CircRNAs are resistant to RNase R digestion, confirming the circular topology of the artificial circRNA. All constructs were expressed from the tetracycline-inducible promoter (TREp). The asterisk (*) marks a putative mechanically linearized product of the corresponding circRNA. Linear/circRNA ratio quantification was performed by digital densitometry using ImageJ.

C Quantification by different methods of the linear and circular isoforms from RNase R untreated samples of HEK293T cells (naturally lacking miR124) expressing the indicated constructs. Top panels: digital densitometry quantification of Northern blot bands by ImageJ. Bottom panels: RT-qPCR quantification using primers specific for the circular isoform or for both the linear and circular isoforms combined (Total output) as depicted in Figure 2A. In red, the mean is indicated. n = 2 culture wells (from 1 experiment) of HEK293T cells for each condition.

D RT-qPCR measurement of the circRNA-to-linear RNA ratio of RNase R treated vs. untreated samples in cortical neurons or HEK293T cells using divergent primers depicted in Figure 2. Values reflect relative rather than absolute ratios of the measured isoforms. n = 3 culture wells (from 1 primary culture/experiment) of each cell type and for each condition.

E Top: Agarose gel showing triplicates of the RT-qPCR amplicons obtained with divergent primers against the backsplicing junction of the artificial circRNA, after retro-transcription with two different reverse transcriptases (MMLV-RT & Superscript II) in order to rule out artifacts due to template switching during cDNA synthesis. Bottom: Sanger sequencing of the amplicons shown above confirming backsplicing junction in HEK293 cells.

F Left: Northern blot analysis of in HEK293T cells expressing the indicated constructs bearing either perfect or seed-mutant miR-92a binding sites. Right: digital densitometry quantification of Northern blot bands by ImageJ.

In (F), data are presented as mean ± SEM. Statistical significance was determined by unpaired Student's *t tests* (ns: $p > 0.05$, *: $p <= 0.05$, **: $p <= 0.01$, ***: $p <= 0.001$, ****: $p <= 0.0001$).

# Supplementary Figure 3

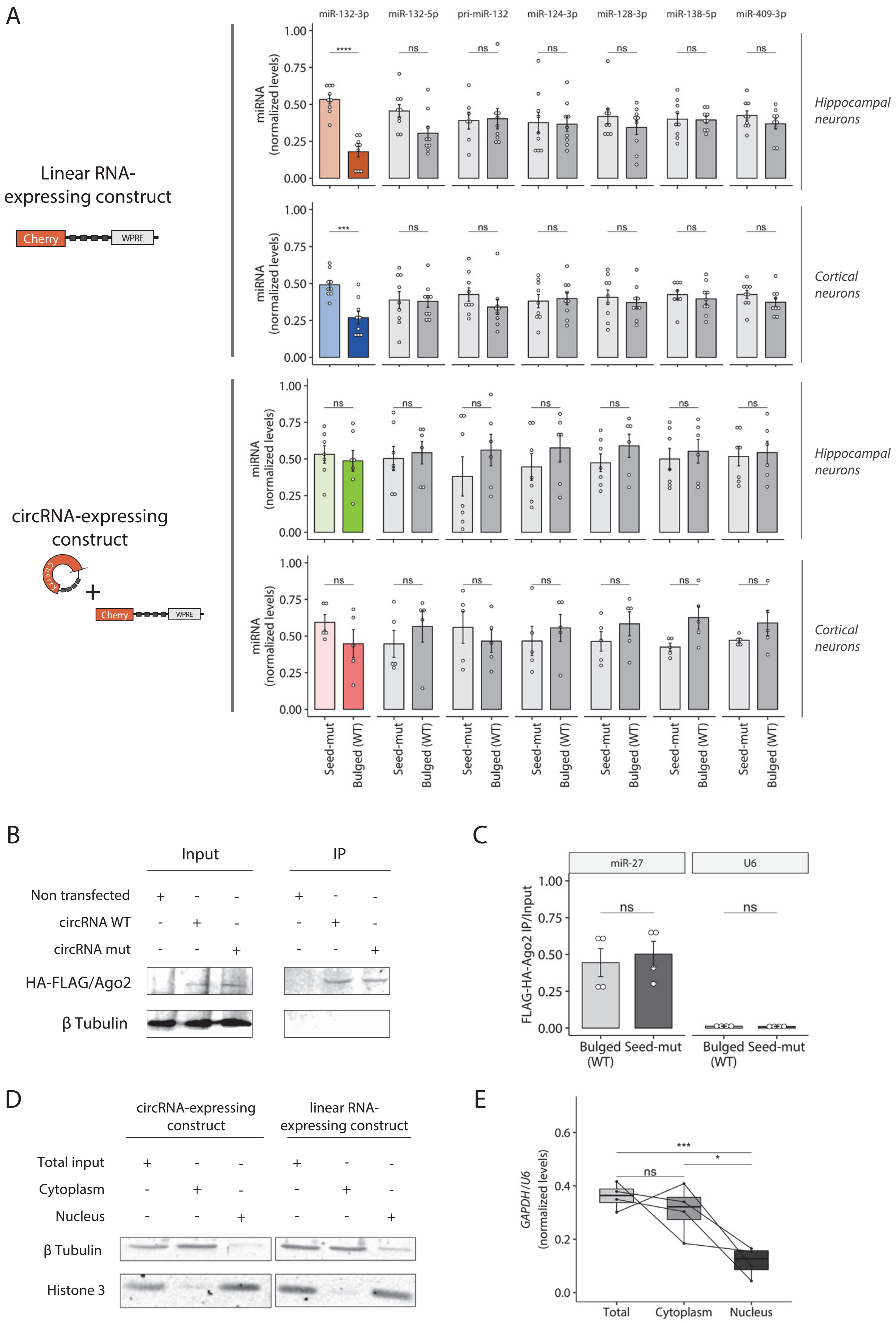

**Supplementary Figure 3.**

**TDMD specificity, HA-FLAG/AGO2 RIP and cellular fractionation quality controls.**

A RT-qPCR quantification of the indicated RNA species confirms specific degradation of miR-132 guide strand (miR-132-3p) and excludes potential transcriptional effects. Levels of miR-132 passenger strand (miR-132-5p), primary transcript (pri-miR-132) and four unrelated miRNAs (miR-124-3p, miR-128-3p, miR-138-5p and miR-409-3p) were normalized to *U6*. n = 9 culture wells (from 3 independent primary cultures) of cortical neurons for each condition; n = 9 culture wells (from 4 independent primary cultures) of hippocampal neurons for each condition. Missing points are failed culture wells/RT-qPCR reactions.

B Representative anti-HA Western Blot from inputs or anti-FLAG IPs in non-transfected HEK-293T cells or cells co-transfected with HA-FLAG/AGO2 and the circRNA-expressing construct bearing bulged (WT) or seed-mutant (mut) miR-132 sites.

C *U6* and miR-27 levels were measured to verify the efficiency of HA-FLAG/AGO2 immunoprecipitation. n = 4 culture wells (from 2 experiments) of HEK293T cells for each condition.

D Representative Western Blot of HEK293T cells transfected with either the circRNA-expressing construct or the linear control RNA, following subcellular fractionation. β-Tubulin and Histone-3 were used as cytoplasm and nucleus markers, respectively.

E Boxplot depicting median values of *GAPDH*/*U6* ratio measured by RT-qPCR confirms proper subcellular fractionation of samples either transfected with the circRNA-expressing construct or the linear control. n = 4 culture wells (from 1 experiment) of HEK293T cells for each condition.

In (A, C), data are presented as mean ± SEM. Statistical significance was determined by unpaired Student's *t tests* (ns: $p > 0.05$, *: $p <= 0.05$, **: $p <= 0.01$, ***: $p <= 0.001$, ****: $p <= 0.0001$). In panel A, Bonferroni's correction for multiple testing was applied.

# Supplementary Figure 4

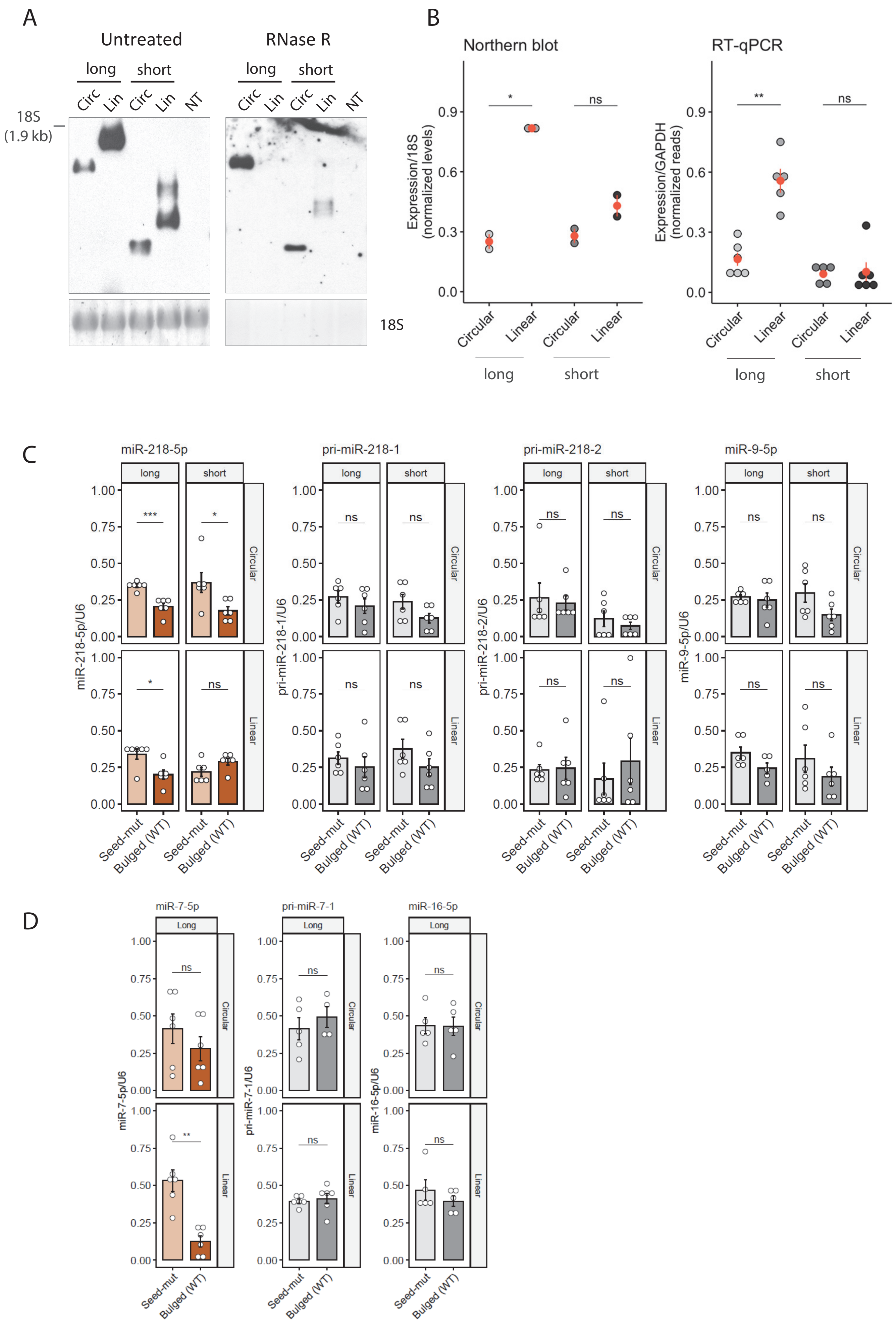

**Supplementary Figure 4.**

**CircRNA-expressing constructs and quality controls.**

A Northern blot analysis of RNase R treated or untreated samples from HEK293T cells expressing the constructs of the indicated length. CircRNAs are resistant to RNase R digestion, confirming the circular topology of the artificial circRNA.

B Quantification by different methods of the linear and circular isoforms from samples of HEK293T cells expressing the indicated constructs. Left: digital densitometry quantification of Northern blot bands by ImageJ. Right: RT-qPCR quantification using primers specific for both the linear and circular isoforms combined (Total output). In red, the mean is indicated.

C RT-qPCR quantification of the indicated RNA species confirms specific degradation of miR-218 guide strand (miR-218-5p) and excluding potential transcriptional effects. Levels of primary transcript (pri-miR-218) and one unrelated miRNA (miR-9-5p) were normalized to *U6*. n = 6 culture wells (from 2 independent experiments) of HEK293T cells for each condition.

D RT-qPCR quantification of the indicated RNA species confirms specific degradation of miR-7 guide strand (miR-7-5p) and excluding potential transcriptional effects. Levels of the main primary transcript (pri-miR-7-1) and one unrelated miRNA (miR-16-5p) were normalized to U6. n = 6 culture wells (from 2 independent experiments) of HEK293T cells for each condition.

Data are presented as mean ± SEM. Statistical significance was determined by unpaired Student's *t tests* (ns: p > 0.05, *: p <= 0.05, **: p <= 0.01, ***: p <= 0.001, ****: p <= 0.0001).

# Supplementary Figure 5

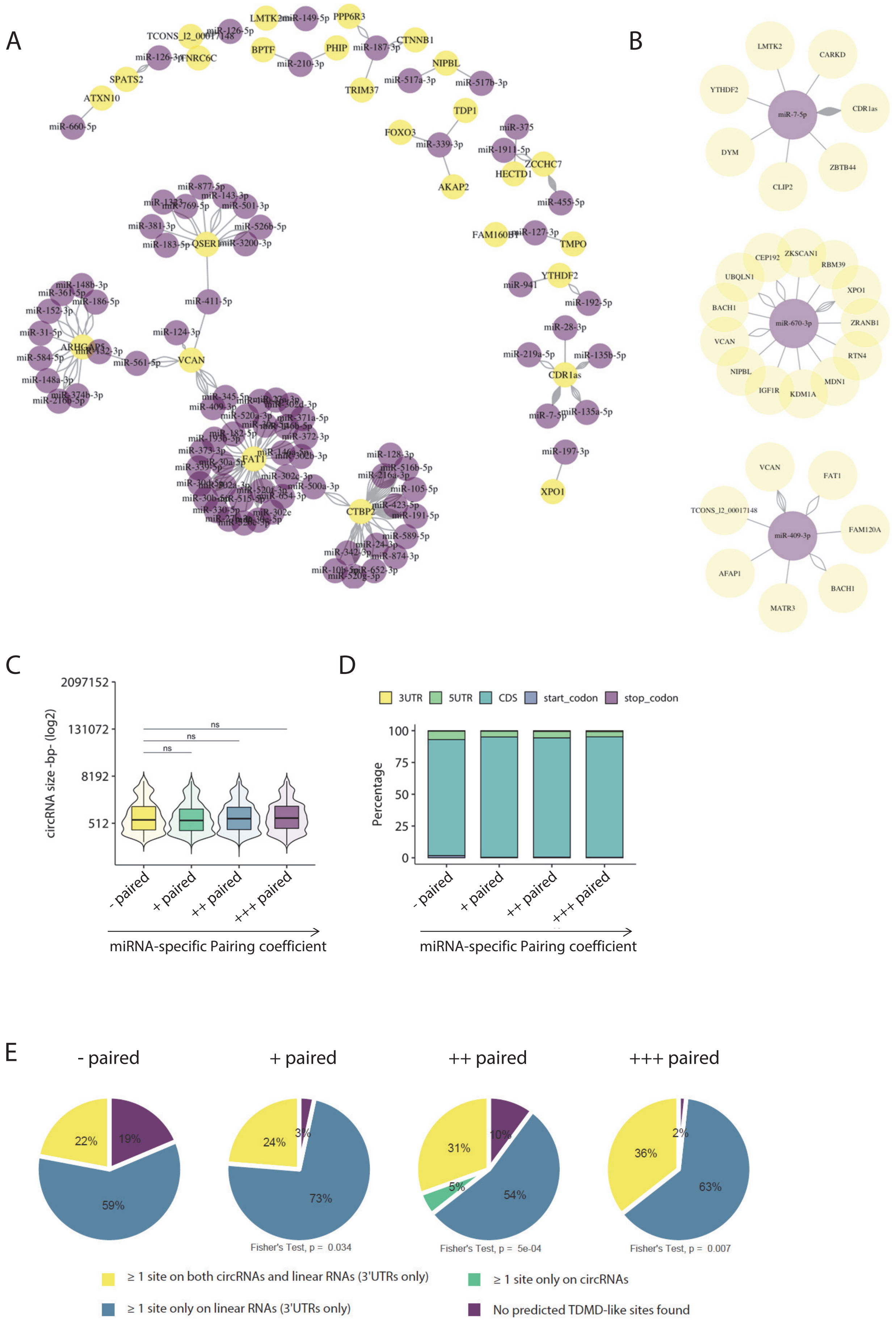

**Supplementary Figure 5.**

**Illustrations of the predicted circRNA-miRNA networks and genomic features of the potentially involved circRNAs.**

A Network diagram of biochemically supported interactions between circRNAs and miRNAs. Depicted are the interactions involving the seven most “pairing” circRNAs.

B Network diagram showing biochemically supported circRNA-miRNA interactions for miR-7 and two other miRNAs with similar “pairing” coefficient (miR-670-3p and miR-409-3p). For all three examples, the stoichiometries (i.e. ratios of circRNA-binding-site:miRNA) are similar, with CDR1as making the largest contribution for miRNA-7. MiR-7 and miR-409 are also a demonstrated case and strong candidate to undergo of TDMD, respectively (see main text).

C Boxplot showing the size of circRNAs (log2 of the base pairs) that interact with miRNAs from each of the different pairing quartiles estimated from data of hESC H9 into forebrain (FB) neuron progenitor cell differentiation (Chen *et al*, 2015; Zhang *et al*, 2016). Shown are Wilcoxon rank sum test p-values (corrected with the Hochberg method for multiple comparisons) between the least paired and the remaining groups (ns: $p > 0.05$).

D Barplot representing the percentages of overlapping genomic regions (CDS, 5’UTR, 3’UTR, etc) giving rise to the circRNAs with predicted binding sites against miRNAs within different quartiles of miRNA-specific Pairing coefficient. No enrichment of genomics features for circRNAs interacting with miRNAs within the different quartiles was observed (Pearson's Chi-squared test p-value = 0.998).

E Pie charts showing the proportion of predicted linear RNA- and circRNA-miRNA interactions involving at least one predicted TDMD-like site against miRNAs within quartiles of increasing miRNA-specific Pairing coefficient. Only 3’-UTRs within linear RNAs of protein-coding genes were considered for this analysis. Shown are the p-values for the Fisher’s Exact test between each quartile and the least paired quartile (“- paired”).

# Supplementary Figure 6

A

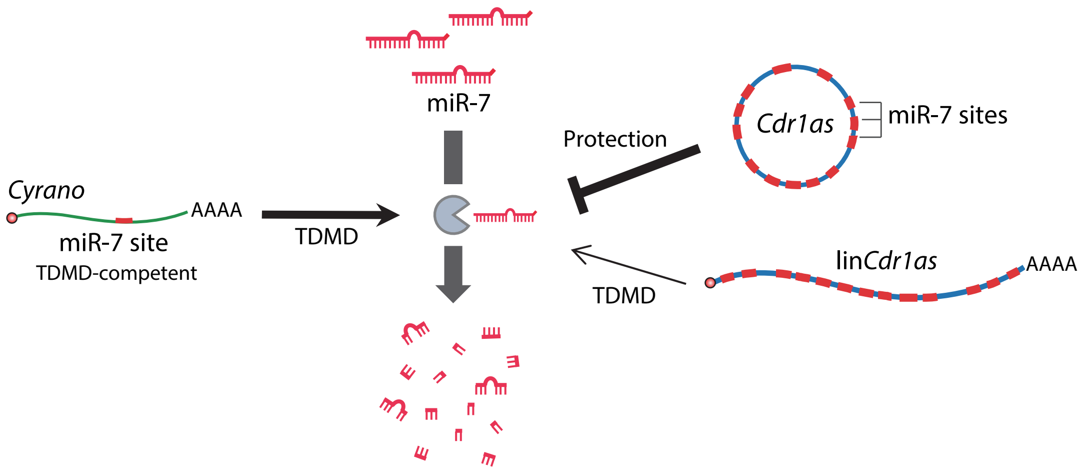


Model for the topology-dependent effect of Cdr1as on miR-7.

B

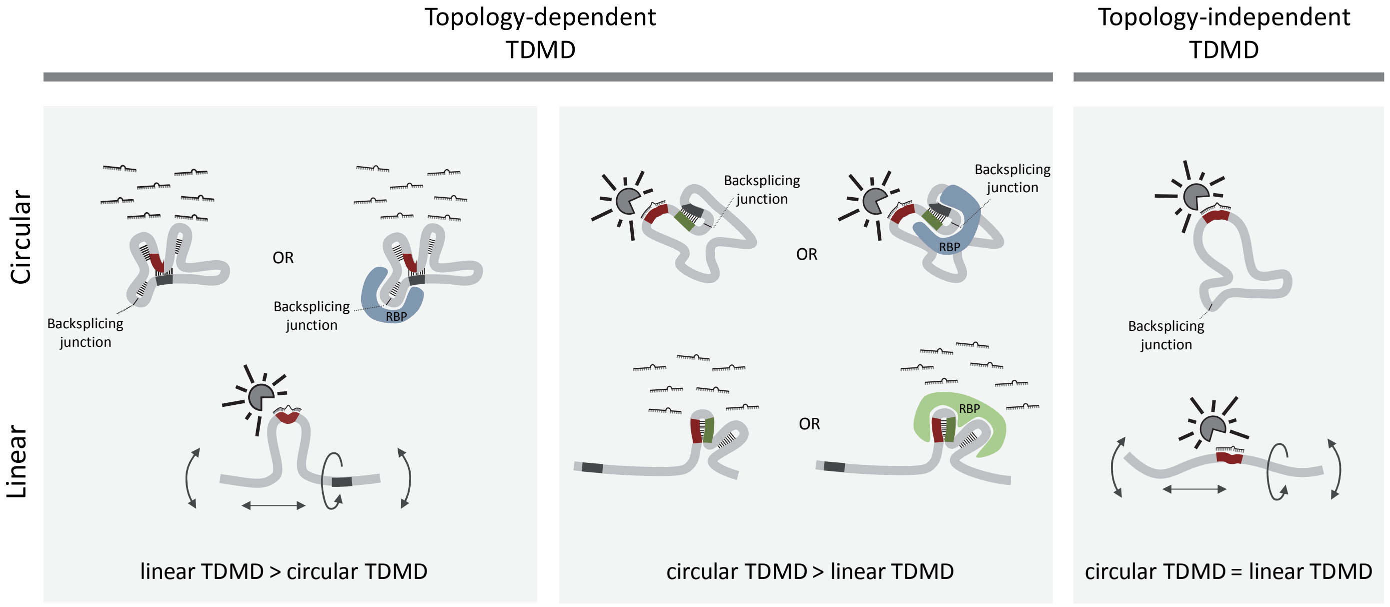

**Supplementary Figure 6.**

A Model for the topology-dependent effect of Cdr1as on miR-7.

**B** Model for the topology-dependent effects of targets on TDMD.

# Supplementary Figure 7

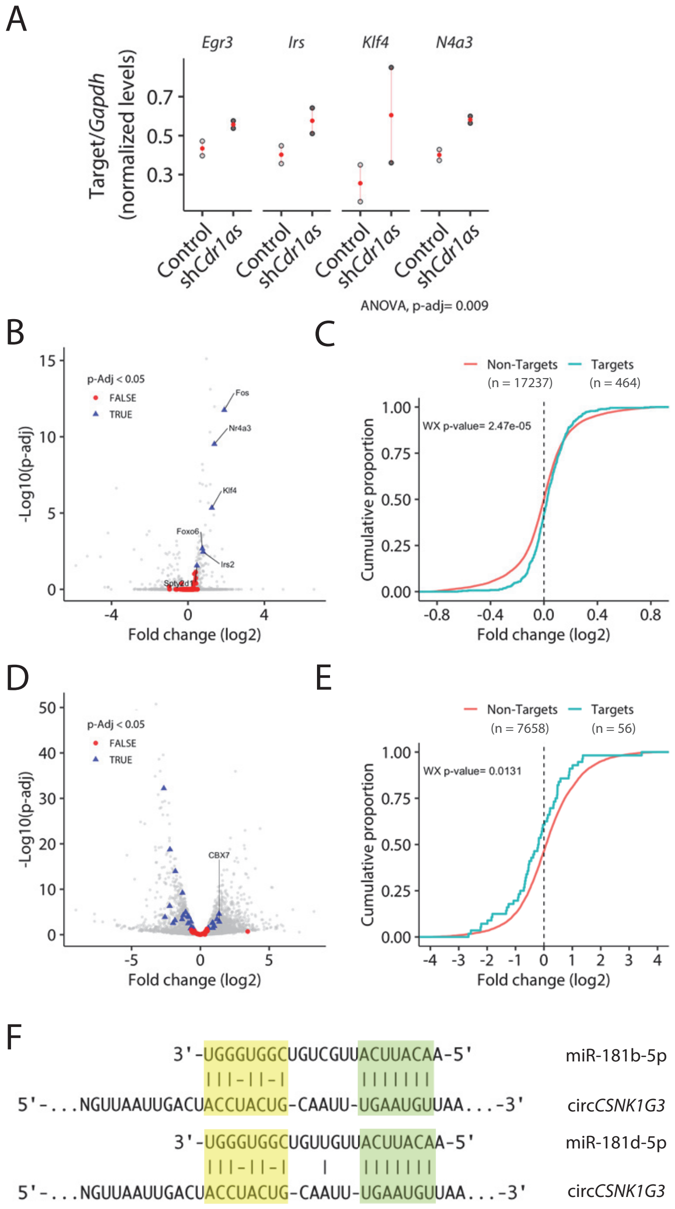

**Supplementary Figure 7.**

**MiRNA stabilization by circRNAs leads to contradictory effects on the overall target silencing in different systems.**

A Abundance of four validated miR-7 targets, measured by RT-qPCR upon Cdr1as knockdown in cortical primary neurons. As previously reported, all four targets were upregulated upon CDR1as KO relative to control (Piwecka et al, 2017). In red, the mean and its standard error (SEM).

B-C Re-analysis of data from Piwecka et al. 2017 confirming that miR-7 predicted targets are significantly upregulated in mouse cortex upon CDR1as knockout. (B) Volcano plot depicting fold-changes (log2) of miR-7 predicted targets in red (p-Adj > 0.05) or blue (p-Adj < 0.05) and non-targets in grey. (C) Fold change (log2) distribution of predicted miR-7-5p targets compared to the background (Non-Targets) in cortex of Cdr1as KO vs. WT mice. Wilcoxon–Mann–Whitney (WX) test p-values are shown.

D-E Re-analysis of data from Chen et al. 2019 to measure average effects on miR-181b/d predicted targets in circCSNK1G3 KD vs. WT PC-3 prostate cancer cells. (D) Volcano plot depicting fold changes (log2) of miR-181b/d predicted targets in red (p-Adj > 0.05) or blue (p-Adj < 0.05) and non-targets in grey. Note that in opposition to the general trend, CBX7 abundance is upregulated upon circCSNK1G3 knockdown, which recapitulates published results (Chen et al, 2019). (E) Fold change (log2) distribution of predicted miR-181b/d targets compared to the background (Non-Targets) in circCSNK1G3 KD vs. WT PC-3 prostate cancer cells. Wilcoxon–Mann–Whitney (WX) test p-values are shown.

F ScanMiR alignment of the miR-181b/d predicted sites on circCSNK1G3 shows extensive 3’ end complementarity.

Fold changes (log2) and p-Adj values were calculated using the DESeq2 package in R.
